# Differential behaviour of normal, transformed and Fanconi's anemia lymphoblastoid cells to modeled microgravity

**DOI:** 10.1186/1423-0127-17-63

**Published:** 2010-07-28

**Authors:** Paola Cuccarolo, Francesca Barbieri, Monica Sancandi, Silvia Viaggi, Paolo Degan

**Affiliations:** 1 Department of Epidemiology, Prevention and Special Functions, National Institute for Cancer Research (IST), Genova, Italy; 2 Department of Biology, University of Genova, Genova, Italy; 3 Department of Advanced Diagnostic Technologies, National Institute for Cancer Research (IST), Genova, Italy; 4 Current Address: Department of Internal Medicine, University of Genova, Genova, Italy

## Abstract

**Background:**

Whether microgravity might influence tumour growth and carcinogenesis is still an open issue. It is not clear also if and how normal and transformed cells are differently solicited by microgravity. The present study was designed to verify this issue.

**Methods:**

Two normal, LB and HSC93, and two transformed, Jurkat and 1310, lymphoblast cell lines were used as representative for the two conditions. Two lymphoblast lines from Fanconi's anemia patients group A and C (FA-A and FA-C, respectively), along with their isogenic corrected counterparts (FA-A-cor and FA-C-cor) were also used. Cell lines were evaluated for their proliferative ability, vitality and apoptotic susceptibility upon microgravity exposure in comparison with unexposed cells. Different parameters correlated to energy metabolism, glucose consumption, mitochondrial membrane potential (MMP), intracellular ATP content, red-ox balance and ability of the cells to repair the DNA damage product 8-OHdG induced by the treatment of the cells with 20 mM KBrO_3 _were also evaluated.

**Results:**

Transformed Jurkat and 1310 cells appear resistant to the microgravitational challenge. On the contrary normal LB and HSC93 cells display increased apoptotic susceptibility, shortage of energy storages and reduced ability to cope with oxidative stress. FA-A and FA-C cells appear resistant to microgravity exposure, analogously to transformed cells. FA corrected cells did shown intermediate sensitivity to microgravity exposure suggesting that genetic correction does not completely reverts cellular phenotype.

**Conclusions:**

In the light of the reported results microgravity should be regarded as an harmful condition either when considering normal as well as transformed cells. Modeled microgravity and space-based technology are interesting tools in the biomedicine laboratory and offer an original, useful and unique approach in the study of cellular biochemistry and in the regulation of metabolic pathways.

## Background

We previously reported [1] that the exposure of normal lymphocytes and lymphoblast cells (LB and HSC93) to modeled microgravity is a stressful process. Upon this condition cells experience proliferative inhibition, depletion in intracellular ATP, enhanced susceptibility to treatment with damaging agents and defects in apoptosis and in DNA repair. This condition may thus increase proneness of the cells to malignant transformation.

The senescence-like phenotype [2] in which cells thrive in a state of apparent idleness [3] observed in cells exposed to modeled and real microgravity, is however hiding important changes in the expression of multiple genes. Microgravity has selective effects on cell viability and proliferation [4], on gene transcription, in the stability of the transcripts [5] and in the modulation of the immune response [5,6]. Several studies employed microarray technologies to characterize the gene expression of lymphocytes exposed to modeled and real microgravity [7,8]. The results reports an altered gene expression in pathways deputed to defense against oxidative stress, immune response, control of apoptosis, cell cycle and tumor suppression. Changes in DNA damage susceptibility [9], differentiation, membrane and surface morphology or cytoskeletal architecture [10] were also reported in lymphocytes, promyelocytes and macrophages. Jurkat T-cells flown on STS95 used for gene expression screening [11] also documented an altered expression of the genes that regulate cell growth, metabolism, signal transduction, adhesion, transcription, apoptosis, and tumor suppression. Melanoma cells exposed to simulated microgravity displayed an altered growth and an increased in the melanine production [12]. Subcutaneous inoculation of these cells in C57BL/6 mices, their syngenic hosts, resulted in efficient tumor induction. This enhanced melanine production suggests that microgravity may affect tumor growth and may drive the selection of a highly tumorigenic cell clone showing increased invasive properties.

The question whether exposure to microgravity might influence tumour growth and carcinogenesis is still an open issue notwithstanding the number of publications devoted to this subject. It is not clear yet if and how normal and transformed cells are differently solicited by microgravity. The study presented here was consequently designed to verify how normal and transformed cell lines respond to exposure to modeled microgravity. We therefore used two normal, LB and HSC93, and two transformed, Jurkat and 1310, lymphoblast cell lines as representative of these two conditions.

Four more cell lines were also employed. FA-A and FA-C cells are two lymphoblast lines established from two Fanconi's anemia (FA) patients. The cells belong, respectively, to complementation groups A and C. The other two lines, FA-A-cor and FA-C-cor are the isogenic corrected correspondent of FA-A and FA-C. FA is a genetic disease associated with a severe pathological condition in the child. The disease is inherited as an autosomic recessive character and its poor prognosis is often related to pancytopenia, bone marrow failure and increased risk to malignancies [13]. While a complete review of the pathophysiological characteristics of the disease are beyond the goals of this paper (for review please refer [14-17]) and while the ultimate biochemical defects underlying FA are not yet completely characterized and the definition of the FA condition still defy complete understanding, our purpose was to employ FA cells as a well established model for a cancer prone disease [18]. Abrogation of the FA pathway and mutations in any of the FA genes results in complex changes in cellular phenotype, biochemistry and metabolism. Such complexity suggests a hierarchically elevated position for this pathway. We were therefore interested to study the behavior of FA cell lines toward microgravity exposure.

## Methods

### Cell lines, Proliferation and Apoptosis

Lymphoblast cell lines LB and HSC93 are normal B and T, respectively, human lymphocytes immortalised with EBV. Jurkat and 1310 are established naturally transformed T-lymphocytes cell lines. FA-A (EUFA-471-L) and FA-C (HSC 536) are lymphoblast cells derived by two FA patients. FA-A cells belongs to complementation group A, and FA-C cells to group C. FA-A-cor and FA-C-cor are spontaneous in vitro revertant from cell lines FA-A and FA-C, respectively [19]. Cell lines are maintained in culture in RPMI 1640 medium supplemented with 10% FCS, 25 mM Hepes and 2 mM L-Glutamine at 37°C at 5% CO_2_. In the various experiment reported here below cells were grown, treated and analysed under identical conditions except for the absence or presence of microgravity. Cell growth was monitored with a BrdU detection kit (Millipore, MA, USA) and proliferation was calculated after quantification of the respective doubling time for each cell line. Cell viability was determined by the trypan blue dye exclusion test. Cell cycle analysis and sub-G1 cell fraction were calculated after FACScan (Beckton Dickinson) analysis. Cells were stained with propidium iodide and 20.000 events were collected from each sample before ModFit analysis.

### Microgravity exposure and cell treatments

Microgravity was accomplished by a random position machine (RPM) machine (Dutch Space, Leiden, NL) located in a temperature controlled room. The RPM [20] is a laboratory instrument designed to randomly change the position of an accommodated biological experiment in 3-dimensional space. The lay-out of the RPM consists of two cardanic frames and one experiment platform (Additional file 1). The frames and the platform are driven by means of belts and two electro-motors.

The RPM is computer managed and a dedicated software permits the settings for modeled microgravity at the value of choice. Rotation rate ω and geometrical distance from the centre of rotation (R) yield 'g-contours', through *g*_i _= ω2R/*g*_0 _(*g*_0 _= 9.81 m/s2), that provide guidelines for the design and lay-out of experiment packages and for the interpretation of the experimental results [20]. Routinary conditions employed in our experiments sets *g *below 0.005 m/s^2^. In the conditions employed in the experiments reported below cells were exposed continuously in the RPM for 24 hours. Eventual exposure to KBrO_3 _(20 mM, 30 min., 37°C, in complete medium) was performed at the end of the exposure schedule.

### Glucose, PARP, ATP, TBARS and protein quantification

The concentration of glucose present in the cell medium was measured with a commercial assay kit (BioVision Glucose assay kit, BioVision, Mountain View, California, USA). Glucose content per cell (mg/ml/cell) was measured in aliquots taken from the culture medium during the different phases of the experiments. PARP activity (pmol/min μg DNA) was determined by quantification of labelled ADP-ribose resulting after (^32^P)NAD (5 μCi/nmol) incorporation into acid insoluble material [21]. In the assay kit employed intracellular ATP (Sigma Chem. Co., St Loius, LO, USA) is measured after the concomitant conversion of ATP to ADP through NADH oxidation to NAD. This reaction is followed by the decrease in the absorbance at 340 nM which is proportional to the amount of the ATP transformed to ADP (μmol/10^6 ^cells). A measure of a general oxidative stress was performed by quantification of lipid peroxides as thiobarbituric acid reactive substances (TBARS) in cell extracts by mean of an assay kit which employs the formation of the spectrophotometrically quantifiable MDA-TBA complex (Cayman Chem. Co., Ann Arbor, MI, USA). Quantification of the protein content in cell extracts was performed according to the BCA assay kit (Pierce Chem. Co., Indianapolis, IN, USA).

### Mitochondrial Membrane Potential (MMP)

The lipophilic cation 5,5',6,6'tetrachloro-1,1',3,3'-tetraethylbenzimidazol- carbocyanine iodide (JC-1; Sigma Chem. Co, St Louis, LO, USA) was used to detect variations in mitochondrial membrane potential (MMP) [22]. When this dye is taken inside mitochondria its membrane potential is measured by quantifying light emission in the range 500-652 nm since JC-1 fluorescence changes reversibly from green to orange as membrane potentials increases. Aliquots of cell suspension are incubated 10 min. at room temperature in complete culture medium in presence of the dye (10 μg/ml) in dark. Following cell wash in PBS fluorescence emission was measured with FACS as reported above.

### Quantification of DNA repair by 8-OHdG removal

8-OHdG content was quantified in DNA extracted from microgravity exposed and untreated cells [23]. Purified DNA was digested to nucleosides by Nuclease P1 and Alkaline Phosphatase. Aliquots of the nucleosides mix are injected in a C-18 HPLC (Beckman System Gold, Beckman Coulter, Inc, Fullerton, CA, USA) column (Supelco, Bellafonte, PA, USA) flown isocratically (5% MeOH, 95% 50 mM Potassium Phosphate, pH 5,2). The analytical column (15 × 0.46 cm) is coupled with a guard C-18-DB cartridge (Supelco). 8-OHdG in the sample is quantified by electrochemical detection after elution through an ESA 5011A analytical cell (ESA, Chelmsford, MA, USA). Unmodified nucleosides are quantified after UV elution through a diode array detector (Beckman Coulter Inc., Fullerton, CA, USA). Sample analysis is accomplished by the Karat software (Beckman). The time course removal of 8-OHdG of cells exposed to microgravity or in unexposed controls is followed after treatment with 10 mM KBrO_3_.

### Statistical analysis

Data were analysed by one-way ANOVA and unpaired two-tail Student's *t*-test using InStat software. Data are from at least three independent experiments. Standard deviation of the mean (± SD) are reported in the figures as error bars.

## Results

### Cell growth, Proliferation and apoptosis

**Figure 1:**
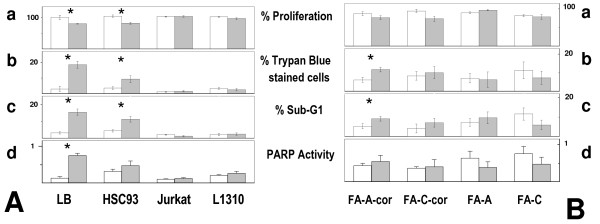
**Growth and vitality in ground unexposed (open columns) or microgravity exposed (close columns) cells**. **A**: LB, HSC93, Jurkat and 1310 cells. **B**: FA-A-cor, FA-C-cor, FA-A and FA-C cells. **Panel a**: Proliferative ability of cells by BrdU assay. Proliferation of LB cells growing in normal conditions was used as reference for the other cell lines. **Panel b**: Percentage of dying cells after trypan blue staining. **Panel c**: Percentage of apoptotic cells after sub-G1 peak quantification in flow cytometry. **Panel d**: PARP activity fluctuactions after autoradiography of cells incubated with (^32^P)NAD.

Figure 1 reports values for various vitality parameters of the cell lines employed in the study. For an easier visualization the values reported are percentages with reference of the value for LB cells in standard conditions. Data are displayed on two separate panels: LB, HSC93, Jurkat and 1310 cell lines are on panel A, and FA-A-cor, FA-C-cor, FA-A, FA-C lines are on panel B.

Proliferation in LB cells (Figure 1A, a) significantly decreased from 100 ± 6.7 to 80.8 ± 1.1% after exposure to microgravity. HSC93 cells behave similarly showing a decrease from 104,80 to 84.3 ± 3.3. No apparent proliferative inhibition was seen for Jurkat and 1310 cells. Among the four FA cell lines, (Figure 1B, a) only FA-C-cor cells displayed a significant decrease in proliferation (from 94.5 ± 4.4 to 73.5 ± 6.7).

According to the trypan blue exclusion test (Figure 1A, b and Figure 1B, b) and the sub-G1 fraction quantification (Figure 1A, c and Figure 1B, c) only LB, HSC93 and FA-A-cor cells displayed a significant increase in cellular mortality upon microgravity exposure.

Poly(ADP-ribose)polymerase (PARP) activity (Figure 1A, d and Figure 1B, d), a stress response activity generally correlated with DNA damage, was significantly increased only in LB cells, whereas HSC93 and FA-A-cor cells displays a non significant increase, after exposure to modeled microgravity.

In conclusion, though at variable degrees, the only cell lines significantly affected by exposure to microgravity are LB and HSC93 and, though at a lesser extent, FA-A-cor and FA-C-cor cells.

### Mitochondria Membrane Potential (MMP)

**Figure 2:**
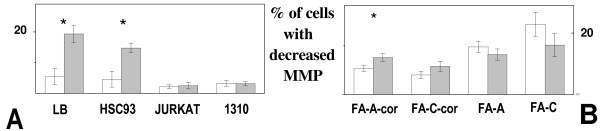
**Variations in MMP in ground unexposed (open columns) or microgravity exposed (close columns) cells**. **A**: LB, HSC93, Jurkat and 1310 cells. **B**: FA-A-cor, FA-C-cor, FA-A and FA-C cells.

Quantification of MMP gives clues on mitochondrial functionality. An elevated polarization of the mitochondrial membrane is related to an efficient electron transport system and an efficient generation of ATP, through oxidative phosphorilation. A decrease in membrane polarization is suggestive of a pre-apoptotic condition. In control conditions LB, HSC93, Jurkat and 1310 cells displays an elevated mitochondrial membrane polarization (Figure 2A). In consequence of microgravity exposure the fraction of LB and HSC93 cells with depolarized mitochondria increased significantly (3.3 and 4.6 folds, for LB and HSC93, respectively). Jurkat and 1310 cells, which display a remarkable low level of depolarized mitochondria in the unexposed condition, were almost unaffected by microgravity. On the contrary all the four FA cell lines displayed high percentages of depolarized mitochondria already when unexposed (Figure 2B). Among these cell lines FA-A and FA-C cells displayed the highest values (17.07 ± 2.03 and 25.2 ± 4.3, respectively). Microgravity exposure induces further decrease in MMP in FA-A-cor and FA-C-cor while in FA-A and FA-C cells the exposure results in an increase in MMP.

In conclusion microgravity induces a significant decrease in MMP in LB, HSC93 and, to a lesser extent, in FA-A-cor cells.

### Glucose Consumption

**Figure 3:**
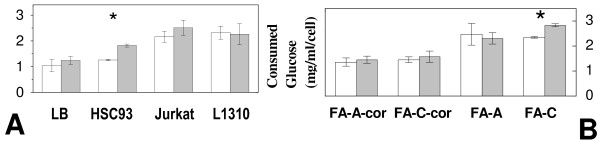
**Glucose consumption (mg/ml/cell) in ground unexposed (open columns) or microgravity exposed (close columns) cells**. **A**: LB, HSC93, Jurkat and 1310 cells. **B**: FA-A-cor, FA-C-cor, FA-A and FA-C cells.

In LB and HSC93 cells growing in normal conditions glucose consumption was quantified, respectively, at 1.04 ± 0.23 and 1.26 ± 0.03 mg/ml/cell (Figure 3A). Basal glucose consumption was about two fold higher in Jurkat and 1310 cells. In FA-A-cor and FA-C-cor cells glucose consumption (Figure 3B) was quantified, respectively, at 1.32 ± 0.16 and 1.41 ± 0.11, where FA-A and FA-C cells display higher values, very close to those measured in Jurkat and 1310 cells. Upon microgravity exposure significant increase in glucose consumption was seen only in HSC93 (Figure 3A) and FA-C (Figure 3B) cells. Glucose consumption is related to cellular proliferation and gives clues on the ability of the cells to rely preferentially on an oxidative rather than a glycolytic metabolism. This condition, known as the 'Warburg effect', is commonly found in cells undergoing transformation [24]. This strategy allows the maintenance of an adequate energy production, and limits excessive oxidative stress and hypoxia thus inhibiting inflammatory processes. An increase in glucose consumption was also been reported in cells exposed to microgravity [25] in relationship with the senescence-like condition reported above [2]. In our hands however glucose consumption is more elevated in Jurkat, 1310, FA-A and FA-C cells regardless microgravity exposure. In conclusion then it is the genetic background rather than microgravity that affects glucose metabolism.

### ATP

**Figure 4:**
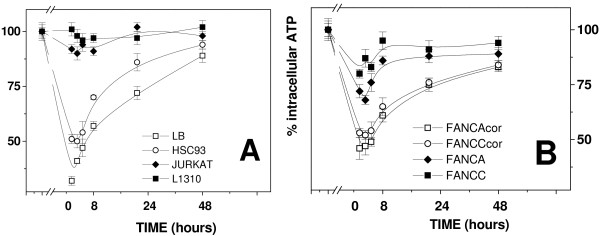
**Recovery in intracellular ATP in ground unexposed (solid symbols) or microgravity exposed (open symbols) cells**. **A**: LB, HSC93, Jurkat and 1310 cells. **B**: FA-A-cor, FA-C-cor, FA-A and FA-C cells. The content of ATP in the various cell lines before exposure to microgravity was normalized to 100%.

Figure 4 reports the time course recovery for intracellular ATP during the 24 hours after the exposure to microgravity. Basal values for ATP in the unexposed cell lines were normalized to the value of LB cells (3.69 ± 0.51 μmol/10^6 ^cells), set as the 100%. Intracellular ATP level measured in the various cell lines, before microgravity exposure were: 83.46% for HSC93, 111.65% for Jurkat, 97.01% for 1310, 94.03% for FA-A-cor, 87.80% for FA-C-cor, 107.3% for FA-A and 79.94% for FA-C,

Exposure to microgravity results in a dramatic drop in intracellular ATP content. Measures taken just at the end of the 24 hours exposure in the RPM showed that in LB cells intracellular ATP went down to 32.4 ± 5.3% of the value in the unexposed control (Figure 4A). A similar decrease was measured also in HSC93 cells (51.6 ± 6.3%). On the contrary Jurkat and 1310 cells did show almost no decrease. Figure 4B displays results obtained for FA cells. A significant decrease in intracellular ATP was measured in FA-A-cor and FA-C-cor cells (52.19 ± 4.16% and 47.53 ± 3.27% respectively). The decrease in ATP level was much lower in FA-A and FA-C cells (72.4 ± 4.3% and 82.1 ± 2.1%, respectively). Recovery to basal level was almost complete in 10 hours for FA-A and FA-C cell lines while it required 24 or more hours for LB, HSC93, FA-A-cor and FA-C-cor.

In conclusion microgravity strongly affects intracellular ATP production at least among LB, HSC93, FA-A-cor and FA-C-cor cells. The two naturally transformed Jurkat and 1310 cells appear unsusceptible to the treatment. The two mutants FA cell lines, FA-A and FA-C, display a reduced susceptibility to the treatment.

### Oxidative Stress: TBARS and 8-OHdG repair

A reliable measure of oxidative stress is the quantification of 8-hydroxy-2'-deoxyguanosine (8-OHdG), a good marker of oxidative DNA damage [26]. An increased oxidative DNA damage is often associated with the presence of genetic defects, altered metabolic fitness and unhealthy state. It is also commonly accepted that microgravity exposure does elicits an inflammation-like reaction. In these conditions the study of 8-OHdG repair kinetic may give clues whether microgravity does affects cell efficiency to cope with this stress. In our hands however no significant increase of 8-OHdG over the basal level was seen after exposure of the cells to microgravity, as reported on Table 1 (compare lanes 1 and 2).

**Table 1:** 8-OHdG induction following microgravity and/or KBrO_3 _treatment.

		**8-OHdG (mol 8-OHdG/10**^**6**^**mol dG)**			
		
	***lane***	***1***	***2***	***3***	***4***
	KBrO_3_	-	-	+	+
	microgravity	-	+	-	+
LB		0.54	0.59	1.61	1.86
HSC93		0.63	0.52	2.07	1.76
Jurkat		0.68	0.74	2.43	2.69
1310		0.41	0.36	2.19	2.44
FA-A-cor		0.84	0.67	1.97	2.51
FA-C-cor		0.76	0.73	2.87	3.26
FA-A		1.04	0.77	2.32	2.45
FA-C		0.88	0.93	3.21	2.86

**Figure 5:**
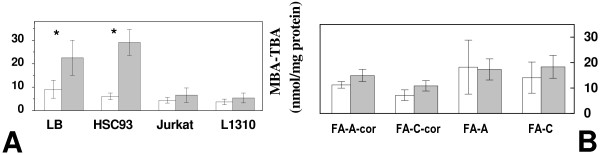
**MBA-TBA (TBARS, nmol/mg protein) quantification in ground unexposed (open columns) or microgravity exposed (close columns) cells**. **A**: LB, HSC93, Jurkat and 1310 cells. **B**: FA-A-cor, FA-C-cor, FA-A and FA-C cells.

We were able, instead, to quantify a basal oxidative stress when measuring thiobarbituric acid reactive substances (TBARS, nmol/mg protein) level, as a measure of free radical mediated lipid damage. As reported on Figure 5A, LB, HSC93, Jurkat and 1310 cell's TBARS levels were 9.1 ± 3.8, 6.1 ± 1.4, 4.4 ± 1.4 and 3.7 ± 1.2, respectively. After microgravity exposure TBARS levels in LB and HSC93 cells increased more than two folds (22.5 ± 7.5 and 29.1 ± 5.5, respectively) while it was without effect in Jurkat and 1310 cells. As shown on Figure 5B basal TBARS levels for FA-A (19.8 ± 11.5), FA-C (15.3 ± 6.6), FA-A-cor (12.2 ± 1.4) and FA-C-cor (7.8 ± 2.3) cells were significantly higher than the values reported for the cells in Figure 5A. So, while TBARS levels did show that these cells sustain a certain unbalance in constitutive red-ox metabolism, this condition appears unaffected by microgravity exposure.

In order to study the eventual microgravity susceptibility of the repair process efficiency within the different cell lines 8-OHdG was induced in cell's DNA by treatment with KBrO_3_. As reported on Table 1 8-OHdG (mol 8-OHdG/10^6^mol dG) was efficiently induced by this treatment (compare lanes 1 and 3). Damage induction was not affected by microgravity exposure as demonstrated by the quantification of 8-OHdG in the two conditions (compare lanes 1 to 2 and 3 to 4).

**Figure 6:**
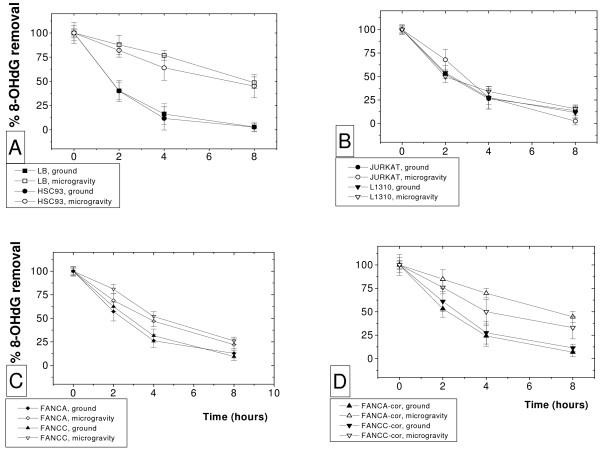
**Time-dependent removal of 8-OHdG in ground unexposed (close symbols) or microgravity exposed (open symbols) cells**. **A**: LB and HSC93 cells. **B**: Jurkat and 1310 cells. **C**: FA-A and FA-C cells. **D**: FA-A-cor and FA-C-cor cells.

Conversely 8-OHdG repair efficiency in LB and HSC93 appear strongly affected by microgravity exposure (Figure 6A). When 50% adduct removal is reached in LB cells in 93 ± 16 minutes, after microgravity exposure the same result was obtained in 463 ± 34 minutes, which roughly means a 5 folds decrease in the efficiency of the process. Almost the same behavior is seen in the HSC93 cells which, upon microgravity exposure, display a decrease in 50% repair efficiency from 108 ± 9 to 346 ± 91 minutes with an overall 3 folds decrease in the efficiency of the process. Jurkat and 1310 lymphoblast cells appear much less affected by microgravity exposure as reported on Figure 6B. In unexposed Jurkat and 1310 cells 50% repair is reached, respectively, in 109 ± 12 and 122 ± 25 minutes. After microgravity 50% repair is reached in 106 ± 18 and 118 ± 23 minutes, respectively, for Jurkat and 1310 cells.

In the case of the four FA cell lines the situation appears a bit more complicate. FA-A and FA-C cells are naturally deficient in 8-OHdG repair efficiency (Figure 6C). In unexposed cells removal of 8-OHdG was very similar in the two mutant FA (Figure 6C) and in the two FA corrected cell lines (Figure 6D). The 50% removal was calculated in 168 ± 49 minutes for FA-A, 186 ± 63 minutes for FA-C, 141 ± 21 for FA-A-cor and 156 ± 33 for FA-C-cor cells). After microgravity exposure a greater decrease in the efficiency of the process is seen in FA-A-cor and FA-C-cor cells (418 ± 73 and 387 ± 41 minutes, respectively) than in FA-A and FA-C cells (223 ± 86 and 251 ± 83 minutes, respectively). While at a lesser extent than in the comparison between LB/HSC93 and Jurkat/1310 cells still a difference in microgravity susceptibility is maintained when comparing repair efficiency of FA-A/FA-C mutant cells and FA-A-cor/FA-C-cor corrected FA cell lines.

## Discussion

### Microgravity exposure differentially affects normal and transformed cells

We show here that transformed Jurkat and 1310 lymphoblast cells are resistant to microgravity exposure in comparison to the normal, EBV immortalised, LB and HSC93 cell lines. This resistance emerges when comparing the performances of these cells to different physiological and metabolic end points. As reported on Figure 1A, LB and HSC93 cells displays a significant decrease in their proliferative ability 24 hour after the exposure to microgravity. Concomitantly to this effect LB and HSC93 cells displays increased trypan blue staining and increased Sub-G1 percentage. LB cells display also a significant increase in PARP activity. None of these parameters appear affected when Jurkat or 1310 cells were subjected to the same protocols of microgravity exposure. Again, as shown on Figure 2A, this same treatment induces significant depolarization of mitochondrial membrane. A decrease in MMP, measured through the JC-1 green fluorescence which typically stains cells with damaged mitochondria, is indicative of an early apoptotic onset. An highly significant increase in the fraction of cells with depolarized mitochondrial membranes is seen when comparing microgravity exposed LB/HSC93 with respect to Jurkat/1310 cells.

The drop in intracellular ATP production may be a possible consequence of the decreased mitochondrial functionality after microgravity exposure (Figure 4A). LB cells display the highest reduction, up to 60%, of the basal ATP level. Recovery to values of the unexposed cells is accomplished during the following 48 hours. Intracellular ATP is apparently unaffected by microgravity exposure in Jurkat and 1310 cells. Interestingly Jurkat and 1310 cells display enhanced basal glucose consumption with respect to LB/HSC93 cells (Figure 3A). An increase in glucose consumption is commonly found in cells undergoing transformation [24], a strategy which allow the maintenance of an adequate energy production and compensate for possible problems related to mitochondrial failure, inflammation, excessive oxidative stress and hypoxia. Intracellular ATP depletion appear inversely proportional to the ability of a given cell line to employ glucose as a cellular fuel suggesting that those cells which rely significantly on glycolysis do perform better. While it has been often reported that oxidative imbalance occurs in response to simulated microgravitational exposure [1,27-29] we were unable to observe the induction of an oxidative DNA damage by microgravity exposure itself. However TBARS level measured before and after microgravity exposure (Figure 5A) did demonstrate a significant increase in oxidation in LB and HSC93 cells but not in Jurkat and 1310 cells. It is then finally important to note that microgravity exposure does not affects the ability to remove 8-OHdG in Jurkat and 1310 cells (Figure 6B) whereas in LB and HSC93 cells (Figure 6A) the same treatment induces, respectively, a five to three folds decrease in the 8-OHdG removal efficiency.

### The effect of microgravity on Fanconi's anemia cells

Ought to the ability of microgravity to interfere, specifically, in the pathways of DNA repair, energy, red-ox balance and apoptosis, as reported above, we were interested to study the behaviour of cells affected by FA in these conditions. FA cells does indeed presents characteristics that are suggestive of transformed cells: elevated basal oxidative stress, DNA repair defects, altered expression of TNF-α, INF-γ and other cytokines [30,31], increased sensitivity to crosslinking agents, chromosomal aberrations and genome instability (reviewed in [15]. We previously reported [19] peculiarities in the FA's energy metabolism and speculate a yet unknown mitochondrial defect in these cells. While anomalous apoptosis was already reported in FA cells [14,32,33] and defective mitochondrial functionality suggested [19,34] recently defects in the cells from the FANCG group were reported [35]. The FA-G protein is localised inside the mitochondria and FANCG mutants displays mitochondria with distorted structures.

FA-A and FA-C cells display a consistent stability to microgravity exposure (Figure 1B). Proliferative ability is maintained in the cell lines and trypan blue staining, percentage of Sub-G1 fraction as well as PARP activity are not significantly affected, with respect to unexposed cells, by microgravity. Also MMP (Figure 2B) is not significantly affected in these conditions.

Microgravity induces depletion in intracellular ATP (Figure 4B) to 72.4 ± 4.3% and 82.1 ± 2.1%, respectively, for FA-A and FA-C cells, which is less dramatic in comparison with the depletion reported for LB and HSC93 cells. Recovery to values of the unexposed cells is accomplished thereafter and both FA-A and FA-C cells display the fastest recovery kinetics. Regardless microgravity exposure both FA cell lines show an increase in glucose utilization (Figure 3B), unlike HSC93 and LB cells, at an extent similar to the Jurkat/1310 cells. As mentioned above the adoption of a strategy which permits to these cells to metabolise glucose with high efficiency strongly suggest that these cells thrive in a metabolic equilibrium far from that of a normal cell [24]. Exposure to microgravity induce a further increase in glucose consumption at least for FA-C cells (Figure 3B).

We finally concentrate on the characterization of the oxidative metabolism in the FA cells. An oxidative imbalance in FA cells is suggested by the high level of TBARS measured in these cells in basal conditions (Figure 5B). However after microgravity exposure TBARS content does increased significantly in LB and HSC93 cells while it does not increase in FA-A/FA-C nor in the two FA-corrected cell lines. While FA cells are defective in repair of oxidatively damaged DNA [36] and a 2-fold slower kinetic of repair of 8-OHdG was found in the comparison between LB and HSC93 cells (please compare panels A, C and D in Figure 6), when these cells were exposed to microgravity the decrease in 8-OHdG repair efficiency found in FA-A and FA-C cells (Figure 6C) was reduced in comparison with what was observed in LB and HSC93 cells (Figure 6A).

## Conclusions

In conclusion microgravity appear able to differentially affect the physiological properties of the exposed cells. While microgravity exposure may be favourable for the growth and survival of the transformed Jurkat and 1310 cells, in normal cells the increased apoptotic susceptibility, the depletion of energy storages and the reduced repair ability, which are probably linked to the downregulation of genes deputed to the control and regulation of these activities, may likely expose these cells to the risk of malignant transformation [9]. Microgravity should then be regarded as a harmful condition for normal as well for transformed cells and tissues. This is an important issue when considering the health risks associated with the exposure to space environment significantly taking into account solely the contribute of microgravity without the influence of radiation.

A second conclusion is that FA-A and FA-C cells are resistant to simulated microgravity, analogously to Jurkat/1310 transformed cells. On the contrary the behavior of the two FA-corrected cell lines appear closer to the behavior of the normal LB/HSC93 cell lines.

Mutant FA cells display an altered metabolism characterized by defects in the pathways that controls energy production and apoptosis. The same metabolic changes that characterize FA resistance to microgravity are those displayed by cells progressing from a normal to a transformed phenotype [24]. We can thus speculate that microgravity appear agonistic with transformation and the resistance to microgravity displayed by the FA cells furtherly underlines their carcinogenic potential.

Modeled microgravity and space-based technology can eventually be regarded as interesting and unique tools in the biomedicine laboratory as they offer an original, useful and unique approach in the study of cellular biochemistry and in the regulation of metabolic pathways. Experiments employing modelled, instead of space-based, microgravity have the advantage to select solely for this stressor without the interfering effect of radiation. Furthermore modeled microgravity experimentation can be realized without constrains in terms of sample amounts, numerosity and size with the advantage to test many different exposure conditions. Experiments can thus be performed with economy and accuracy, conditions that are impossible to realize in space.

## Competing interests

The authors declare that they have no competing interests.

## Authors' contributions

PC, FB and MS prepared and performed all the experimental work presented in the paper. PD performed the HPLC analysis. SV helped with the rationale in the project and discussed it. PD prepared the manuscript.

## Supplementary Material

Additional file 1 **Portrait of the Random Positioning Machine (RPM)**. Portrait of the RPM used to simulate microgravity exposure of the cells. RPM is located in a room that permits temperature control and ad hoc manipulations. Click here for file
