# Supplementary figures and images for: Differential behaviour of normal, transformed and Fanconi's anemia lymphoblastoid cells to modeled microgravity
Source: J Biomed Sci. 2010 Jul 28;17(1):63. doi: 10.1186/1423-0127-17-63 (PMC2916896; doi:10.1186/1423-0127-17-63)

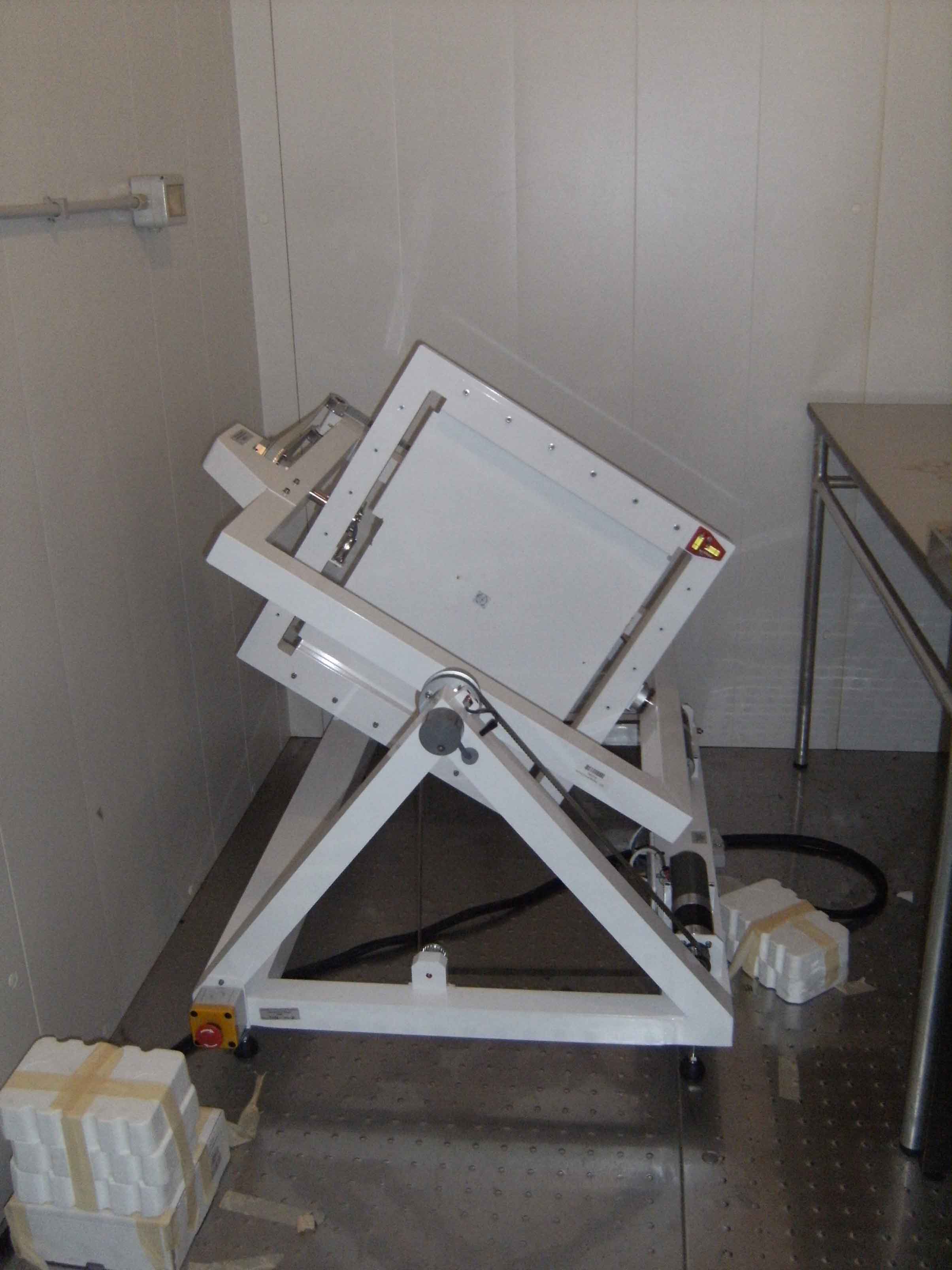

Supplement: Additional file 1 — Portrait of the Random Positioning Machine (RPM). Portrait of the RPM used to simulate microgravity exposure of the cells. RPM is located in a room that permits temperature control and ad hoc manipulations. [file 1423-0127-17-63-S1.JPEG]
